# Energy justice in education sector: The impact of student demographics on elementary and secondary school energy consumption

**DOI:** 10.1016/j.heliyon.2023.e16191

**Published:** 2023-05-11

**Authors:** Zefeng Huang, Zhonghua Gou, Senhong Cai

**Affiliations:** School of Urban Design, Wuhan University, Wuhan, China

**Keywords:** School energy consumption, Student characteristics, School demographics, Learning activities, Energy-saving policies

## Abstract

Reducing school energy costs has become an important issue, while the energy saving should consider different school systems and student backgrounds. This study investigated the impact of student demographics on energy consumption in elementary and secondary schools and explores the difference of energy consumption in different types and levels of school systems. Data were collected from 3672 schools (including 3108 elementary and 564 secondary schools, respectively) in Ontario, Canada. The number of students whose first language is not English, the number of students who receive special education services, the number of school-aged children who live in low-income households, and student learning ability are all inversely proportional to energy consumption; student learning ability has the largest negative impact. The partial correlation between student enrollment and energy consumption has a trend of gradually increasing as the grade levels increase in Catholic elementary schools, Catholic secondary schools, and public secondary schools; however, the correlation shows a gradually decreasing trend with the increase in grade levels in public elementary schools. This study is helpful for policy-makers to clarify the energy implications of various student backgrounds and the energy consumption difference in different types and levels of school systems to facilitate their formulation of effective policies.

## Highlights

• The study revealed the impact of student backgrounds on school energy consumption.
• Student learning ability has the largest negative impact on energy consumption.
• Energy policy should consider the education needs for the disadvantaged groups.
• Differences in energy consumption between different types and levels of school systems were explored.
• It is helpful to formulate effective and just energy-saving policies in school systems.

## 1 Introduction

Schools are at a confluence of global trends and upheavals that have impacts on energy consumption [1]. Energy consumption in school buildings typically accounts for a significant portion of a country’s total energy consumption in the building sector [2,3] and ranks fourth in the United States and the United Kingdom for non-residential buildings [4]. A large amount of energy consumption results in significant energy costs, for example, Canadian schools spend approximately $500 million annually on energy [5], which represents a large percentage of school operating costs. In the coming time, with the widespread emphasis on population quality in various countries, access to education will continue to rise [6,7], and schools as carriers of education are bound to show rapid growth, accompanied by higher energy consumption levels and costs [8]. Although the energy costs of schools are high, they are largely manageable because of the great potential for energy savings in this building type [9,10]. By managing energy more efficiently, schools could reduce energy costs by 15–20% [5], with schools in the UK reducing energy costs and CO_2_ emissions by about 44 million pounds and 625,000 tons per year, respectively [11].

However, the complexity of school buildings has made it difficult to reduce the high energy costs by utilizing traditional energy-efficient means [12], so more studies are needed to help understand the energy consumption of school buildings. Most studies have been conducted on higher education buildings such as universities and colleges. For example, Mahmoodzadeh et al. [13] evaluated the potential energy savings of vertical envelope retrofitting of 49 campus buildings in the University of Victoria, Canada, and first used the heat loss coefficient (HLC) to quantify the thermal performance of the university building envelope. Gui et al. [14] selected 122 university buildings located in Australia and collected their weekly electricity data and detailed space use information, showing that the health sciences was the most energy-intensive discipline, and buildings dedicated to research have the highest energy use intensity. Liu and Ren [15] took the most representative Chinese university building types, such as libraries and dormitories, as examples and established a model of energy-saving design strategies for Chinese university buildings based on green performance analysis. Xu et al. [16] studied 17 buildings on the campus of Southeast University in Nanjing, China and predicted multibuilding energy use by integrating social network analysis (SNA) and artificial neural network (ANN) techniques. Only a few scholars have conducted studies on elementary and secondary schools, which are a critical part of fundamental education. Wang [17] studied the energy consumption of 7 senior high schools, 11 junior high schools, and 5 elementary schools in Taiwan by developing a regression model to determine the energy consumption. Droutsa et al. [18] used energy audit data from approximately 350 school buildings in the Greek national electronic repository to reveal existing conditions and energy use of school buildings and calculated the final energy use intensity and primary energy use intensity, as well as the respective energy classes. Chung and Yeung [19] studied 121 secondary schools in Hong Kong and benchmarked the average energy use and energy use intensity for each school.

Elementary schools, secondary schools, and universities belong to different school systems, and they have different educational purposes, content, activities, methods, and environments, resulting in different levels of energy consumption. At the same time, elementary and secondary schools, which are part of fundamental education, are more universal and extensive than universities. Therefore, given the high volume of research on higher educational energy consumption, it is imperative to conduct corresponding research on elementary and secondary school systems.

Several ambitious energy-saving policies and programs for improving energy consumption in schools are being widely implemented [20]. At the governmental level, the U.S. Department of Energy released the EnergySmart Schools Program [21], some of the key conclusions of which include a 5%–20% reduction through effective management, maintenance, and operation of physical school equipment and detailed energy policies that should provide guidance for operation and maintenance programs [22]. The Green School Project is a policy of the United States for energy savings that aims to improve the energy and environmental efficiency of existing school buildings [23]. The Bright Schools Program is a California Energy Commission policy that provides specific services to help people retrofit or build new energy-efficient school buildings [24]. In Canada, announced in May 2019, the Climate Action Incentive Fund allocated up to CAD 218 million in Saskatchewan, Manitoba, Ontario, and New Brunswick for projects that reduce energy use and/or greenhouse gas (GHG) emissions. Schools were identified as the priority for 2019–2020 [25]. Building Schools for the Future is a long-term investment and reform policy in England that aims to provide a learning environment that will help transform secondary school students' education by improving the energy efficiency of school buildings and developing a sustainability framework [26]. In addition, at the school level, many well-known universities, including Massachusetts Institute of Technology [27], University of New South Wales [28], University of West England [29], and Yale University [30], have also released energy efficiency and emissions reduction programs for school buildings. They focused on the impact of building materials, building structures, internal building equipment, and the adoption of renewable energy on building energy savings [27,28], and outlined several measures to reduce their carbon emissions by adopting more advanced technologies (e.g., using highly energy-efficient [29] and smart devices [30]) and retrofitting older buildings [27].

In terms of implementation, these existing energy-saving policies and programs are primarily based on new construction, renovation, or maintenance of the school facilities, either through financial subsidies or technological refinement, as a way to improve the school energy efficiency. However, they ignore the potential for energy savings due to the conditions of students and the related educational activities. For schools, these conditions should cover the characteristics of different students and the corresponding learning activities, with attention to disadvantaged groups such as students from low-income families and students from migrant families, but few policy-makers utilize or incorporate them into school system energy policies and programs.

Studies have proven that the physical attributes of the building itself alone cannot explain building energy consumption, since the characteristics of the occupants, as indicators of behavioral patterns, largely impact the energy consumption level, both in residential and non-residential buildings [[31], [32], [33]]. For example, age and income have been found to be factors that have a strong influence on building energy consumption [34]. In school buildings, studies have shown that user awareness is also important to promote energy efficiency in the green building concept [35]. At the same time, students are the main users of energy in schools, and their awareness of energy efficiency may vary depending on their own backgrounds, which requires targeted energy efficiency education that reflects their characteristics in order to promote the effective reduction of energy consumption in schools. Therefore, it is important to explore the impact of students' backgrounds on energy consumption in school buildings.

Overall, there is a lack of research on energy consumption in elementary and secondary school systems, as well as a lack of research to elaborate on the relationships between students' background conditions and school energy consumption characteristics. To fill this gap, this study explores the impact of students' background conditions on school energy consumption. It also analyzes the differences of energy consumption in different types and levels of school systems and explores which grade of students has a greater impact on various types of energy consumption. In addition, this study provides insights into existing school energy-saving policies and programs from the perspective of students' individual characteristics to make more inclusive and just energy policies and programs. The key question for this study to investigate is whether student demographics have an impact on energy consumption in elementary and secondary schools. In addition, if there is an impact, what characteristics of student backgrounds can explain the energy consumption difference. Answering these research questions can help to identify energy saving opportunities in the school sector, especially for developing more meaningful educational programs for future generations.

## 2 Methodology

### 2.1 Study area and data source

The province of Ontario, Canada, located in eastern Canada (41°-57°N, 74°-95°W) and covering an area of approximately 1 million square kilometers, is the most populous of Canada’s ten provinces [36]. This large, ethnically diverse province is composed of immigrants, of whom approximately 65% are of British descent, 5% of French descent, and the rest mostly of Asian and Aboriginal descent. This demographic profile provides the basis and possibility to study the impact of students with different backgrounds on school energy consumption. In addition, the region has long and cold winters, with the lowest temperatures of the year being in January, reaching down to −25 °C in some areas. The summer is slightly warmer, with the highest temperatures occurring in July, ranging from 20 °C to 30 °C in the daytime with high humidity. Therefore, the heating energy demand of school systems in winter is greater than the cooling energy demand in summer.

In this study, the energy use and greenhouse gas emission datasets [37] of schools in Ontario in 2018 and 2019 were first collected through the Ontario Data Catalogue website. The dataset includes school information, total indoor space, weekly average hours, annual electricity consumption, annual natural gas consumption, and annual greenhouse gas emissions for a total of more than 4000 schools in the Ontario area. The energy consumption data have been climate-normalized to eliminate abnormal energy consumption due to different levels of building heat or cooling loads caused by extreme weather in a given year.

Subsequently, this study also collected the school characteristics and student demographics dataset [38] as well as the school enrollment by grade dataset [39] for the three school years 2017–2018, 2018–2019 and 2019–2020 from September to June for over 4000 schools in the Ontario region through this website. The school characteristics and student demographics dataset include school information, school type, school level, enrollment, percentage of students whose first language is not English, percentage of students receiving special education services, percentage of school-aged children who live in low-income households, and student achievements for reading, writing, and mathematics. Among them, the school type includes Catholic school and public school. Their differences mainly lie in the source of students and class size. Catholic school is mainly composed of students who are optimized as Roman Catholic and/or have at least one Roman Catholic parent or guardian, and the class size is slightly smaller than that of public school. The school enrollment by grade dataset includes school information and enrollment by grade data. At the same time, these two datasets mainly include public school systems and Catholic school systems in the Ontario area but do not include other types of school systems, such as private schools, summer and night schools, and adult continuing education day schools.

### 2.2 Data pre-processing

To match school energy use data with school characteristics and student demographic data from different datasets and to meet subsequent analysis needs, this study used school information data to filter out schools that had records in different datasets and deleted some schools with missing energy use data or student demographic data. Finally, through a series of data screenings, 3672 schools were identified as research objects for subsequent analysis.

In this study, the raw data were pre-processed in two ways to enable the data to meet the requirements of the subsequent analysis. First, the raw data using percentage units, such as the percentage of students whose first language is not English, the percentage of students receiving special education services, and the percentage of school-aged children who live in low-income households are relative data; thus, the same percentage in different schools represents different numbers of students. Therefore, it is necessary to first calculate the data on the number of students for each variable by combining the percentage data and the total number of student data in each school.

Second, since the school characteristics, student demographics, and school enrollment by grade dataset are recorded for each school year, the energy use and greenhouse gas emission datasets are recorded in natural years. Therefore, to match the data from different datasets over time, this study averaged the data of the two school years of 2017.9–2018.6 and 2018.9–2019.6 after the above data processing to obtain the average data for 2018 and averaged the data of the two school years of 2018.9–2019.6 and 2019.9–2020.6 after the above data processing to obtain the average data for 2019.

### 2.3 Data dimension reduction

The data of student achievements for reading, writing, and mathematics in the school characteristics and student demographics dataset included grade 3 students achieving the provincial standard in reading, grade 3 students achieving the provincial standard in writing, grade 3 students achieving the provincial standard in mathematics, grade 6 students achieving the provincial standard in reading, grade 6 students achieving the provincial standard in writing, and grade 6 students achieving the provincial standard in mathematics, for a total of six variables. However, there is a high degree of overlap and correlation of information among these variables, which creates many obstacles to subsequent statistical analysis. Therefore, it is necessary to reduce the dimensionality of these original variables without causing a large loss of the information contained in them so that the original variables can be integrated into a few or one comprehensive index to facilitate subsequent data analysis. Factor analysis is a data processing method that can effectively reduce the dimension of data variables and has been widely used [40,41]. This study used SPSS Statistics 24 software for factor analysis. The method is mainly divided into the following three steps: (1) Judge whether the preconditions for factor analysis are met; (2) Determine the number of factors, extracting factors and making the factors have named interpretability; (3) Calculate the factor scores of each original variable.

The first step is to judge whether the preconditions for factor analysis are met, which can be accomplished in various ways, such as calculating the correlation coefficient matrix, calculating the anti-image correlation matrix, performing the Bartlett test of sphericity or performing the Kaiser‒Meyer‒Olkin (KMO) test. This study was carried out using the KMO test, in which the KMO test statistic is an index used to compare simple correlation coefficients and partial correlation coefficients between variables. Its mathematical definition is presented in Equation (1), which shows that the value of the KMO statistic is between 0 and 1. When the sum of squares of simple correlation coefficients among all variables is much larger than the sum of squares of partial correlation coefficients, the KMO value is close to 1. The closer the KMO value is to 1, the stronger the correlation between variables, and the more suitable the original variables are for factor analysis. Conversely, when the KMO value is closer to 0, it means that the correlation between variables is weaker, and the original variables are less suitable for factor analysis. At the same time, Kaiser also gave the commonly used KMO value metrics: 0.9 or more means very suitable, 0.8 means suitable, 0.7 means general, 0.6 means not very suitable, and below 0.5 means extremely unsuitable. After software calculation, the KMO value of the variables used for dimensionality reduction in this study was 0.825, indicating that these variables are suitable for subsequent factor analysis [42].

(1) $$KMO=\frac{\sum_{i}\sum_{j\neq i}r_{ij}^{2}}{\sum_{i}\sum_{j\neq i}r_{ij}^{2}+\sum_{i}\sum_{j\neq i}p_{ij}^{2}}$$

where *r*_*ij*_ is the simple correlation coefficient between variables *x*_*i*_ and variables *x*_*j*_; *p*_*ij*_ is the partial correlation coefficient between variables *x*_*i*_ and variables *x*_*j*_ under the control of the remaining variables.

The second step of determining the number of factors is the main process of factor analysis, which entails extracting the factors and making them have named interpretability. In this study, the number of factors is determined based on the cumulative variance contribution of the factors; the number of eigenvalues is usually chosen as the number of Factors k if the cumulative variance contribution is greater than 0.85. In addition, the key to factor extraction is to solve the factor loading matrix based on the sample data, and the main methods for solving this are principal component analysis based on the principal component model, principal axis factor, maximum likelihood, least squares, and alpha factor extraction. This study uses the principal component analysis method for factor extraction, which is the most widely used method in factor analysis. The key to making the factor interpretable is to rotate the factor loading matrix. There are two rotation methods: orthogonal rotation and oblique rotation. In orthogonal rotation, the axes are always rotated at a vertical angle of 90° so that the new factors remain uncorrelated, while in oblique rotation, the angle in the axes can be any degree, and the new factors are not guaranteed to be uncorrelated with each other. Oblique rotations are usually superior to orthogonal rotations in making the factors interpretable, but at the cost of not guaranteeing the uncorrelation of the factors. Therefore, orthogonal rotation is generally preferred. The orthogonal rotation methods usually include the quadratic maximum method (Quartimax), the variance maximum method (Varimax), and the equivalent maximum method (Equamax); this study adopts the variance maximum method.

The third step in calculating the factor score is the final expression of the factor analysis. Once the factors have been determined, the specific values taken for each original variable on each factor, i.e., the factor score, can be calculated, and the new factor formed can replace the original variables for subsequent data analysis. Equation (2) is the factor score function for the jth factor.

(2) $$F_{j}=\varpi_{j1}x_{1}+\varpi_{j2}x_{2}+\varpi_{j3}x_{3}+\cdots \varpi_{jp}x_{p},j=1,2,3,\cdots k$$

where *x*_1_, *x*_2_, *x*_3_, … *x*_*p*_ are the values of the 1st, 2nd, 3rd, …, pth original variables, respectively; $\varpi_{j1},\varpi_{j2},\varpi_{j3},\cdots \varpi_{jp}$ are the factor value coefficients between the jth factor and the 1st, 2nd, 3rd, …, pth original variables, respectively.

In this study, factor analysis was conducted using SPSS Statistics 24 software, and the results showed that when the number of factors was 2, the cumulative variance contribution was 0.96, which exceeded 0.85. It was considered appropriate to extract 2 factors. Table 1 shows the final results of the factor loading matrix, the rotated factor loading matrix and the factor score coefficient matrix calculated by the software when the number of factors is 2. Based on the rotated factor loading matrix and combined with the original variables, it can be seen that the factor F1 can be named as grade 3 student learning ability and the factor F2 can be named as grade 6 student learning ability. The score function for factor F1 (3) and factor F2 (4) can be derived from the factor score coefficient matrix. In addition, based on the score functions of the two factors, the variance contribution of the two factors (where F1 is 0.487 and F2 is 0.477) is set as the weight, and using the method of calculating the total weighted score of the factors, the comprehensive evaluation index of student learning ability F is derived based on factor F1 (grade 3 student learning ability) and factor F2 (grade 6 student learning ability), and subsequent data analysis is carried out. The Equation for calculating this is shown in Equation (5) below.

(3) F1 = 0.421*X1 + 0.427*X2 + 0.427*X3 − 0.160*X4 − 0.156*X5 − 0.183*X6

(4) F2 = −0.159*X1 − 0.167*X2 − 0.168*X3 + 0.429*X4 + 0.424*X5 + 0.439*X6

(5) F = 0.487/(0.487 + 0.477)*F1 + 0.477/(0.487 + 0.477)*F2

**Table 1:** The statistics of factor loading matrix, rotated factor loading matrix, and factor score coefficient matrix.

	Factor loading matrix		Rotated factor loading matrix		Factor score coefficient matrix	
	Factor 1	Factor 2	Factor 1	Factor 2	Factor 1	Factor 2
Grade 3 Students Achieving the Provincial Standard in Reading (X1)	0.912	−0.386	0.921	0.365	0.421	−0.159
Grade 3 Students Achieving the Provincial Standard in Writing (X2)	0.907	−0.396	0.924	0.354	0.427	−0.167
Grade 3 Students Achieving the Provincial Standard in Mathematics (X3)	0.900	−0.397	0.920	0.348	0.427	−0.168
Grade 6 Students Achieving the Provincial Standard in Reading (X4)	0.906	0.395	0.368	0.917	−0.160	0.429
Grade 6 Students Achieving the Provincial Standard in Writing (X5)	0.903	0.390	0.370	0.911	−0.156	0.424
Grade 6 Students Achieving the Provincial Standard in Mathematics (X6)	0.859	0.418	0.319	0.901	−0.183	0.439

### 2.4 Determination of final variable.

Through the pre-processing of the data and the work on dimensionality reduction as described above, the final selection was made to determine the independent and dependent variables used for this study, as shown in Table 2. The numerical variables, such as total indoor space, weekly average hours, and the number of students, were continuous independent variables, while the two categorical variables, school type and school level, were discrete independent variables. The dependent variables were data on the various types of energy consumption and carbon emissions of the school system building, including electricity consumption, natural gas consumption, and GHG emissions. All the variables (including the independent and dependent variables) can be classified into three types according to their nature: school attributes, student demographic attributes, and energy consumption attributes. The descriptive statistics for each variable are shown in Table 3.

**Table 2:** Final determined independent and dependent variables.

Independent Variable		Dependent Variable
Continuous Independent Variable	Discrete Independent Variable	
Total Indoor Space, Weekly Average Hours, Enrollment, Number of Students Whose First Language Is Not English, Number of Students Receiving Special Education Services, Number of School-Aged Children Who Live in Low-Income Households, Student Learning Ability	School Type, School Level	Electricity Consumption, Natural Gas Consumption, GHG Emissions

**Table 3:** Descriptive statistics for each variable.

	Variable Name	Variable Code	Minimum Value	Maximum Value	Average Value	Standard Error
**School Attributes**	Total Indoor Space (Square feet)	X_1_	2304	592,883	63262.55	49776.949
	Weekly Average Hours (h)	X_2_	30.0	105.0	62.856	16.5588
**Demographic Attributes**	Enrollment	X_3_	50	2103	452	302
	Number of Students Whose First Language Is Not English	X_4_	0	1538	126.85	166.925
	Number of Students Receiving Special Education Services	X_5_	0	520	70.25	62.201
	Number of School-Aged Children Who Live in Low-Income Households	X_6_	0	914	81.42	68.218
	Student Learning Ability	X_7_	24	758	210.35	104.221
**Energy Consumption Attributes**	Electricity Consumption (kWh)	Y_1_	0	3,978,045	377574.93	375616.900
	Natural Gas Consumption (m^3^)	Y_2_	0	1,324,278	68141.65	70285.736
	GHG Emission (kg)	Y_3_	0	2,542,846	143098.80	139349.336

### 2.5 Analysis techniques

This study made full use of statistical methods to analyze the data on the variables identified for this study. Its data analysis process mainly includes three main parts. First, multiple regressions were used to analyze the main factors affecting electricity consumption, natural gas consumption, and GHG emissions in school systems and to further explore the specific impact of these factors on various energy consumption levels. Second, the Mann‒Whitney *U* test was used to compare and analyze the differences of energy consumption in different types and levels of school systems. Finally, under the premise of controlling the impact of other variables on various energy consumption, this study uses partial correlation analysis to further explore which grade of students has a greater impact on various types of energy consumption in different types and levels of school systems.

The Mann‒Whitney *U* test, used in the second part of the data analysis, is a method of inferring whether there is a significant difference in the distribution of two independent samples from the total population without knowing much about the overall distribution and can, therefore, be used to analyze differences in energy consumption across different types and levels of school systems. It is mainly inferred by the study of the average rank of two samples, and its main output parameters include the observed value of the calculated test statistic and the corresponding probability P value. Under a small sample, the Mann‒Whitney U-statistic obeys the Mann‒Whitney distribution, and decisions are made based on the probability P value of the U-statistic, which is shown in Equation (6), while under a large sample, the Mann‒Whitney U-statistic approximately obeys a normal distribution, and decisions are made based on the probability P value of the Z-statistic, which is shown in Equation (7). Because of the large sample size of each type and each level of school system collected in this study, the probability P value of the Z-statistic was used for decision-making.

(6) $$U=W-\frac{1}{2}k\left(k+1\right)$$

(7) $$Z=\frac{U-\frac{1}{2}mn}{\sqrt{\frac{1}{12}mn\left(m+n+1\right)}}$$

where the value of W is Wilcoxon W; k is the sample size of the group where W corresponds to the rank sum.

## 3 Results and analysis

### 3.1 Influencing factors

This study uses multiple regression analysis to establish separate multiple regression models for electricity consumption, natural gas consumption, and GHG emissions with various influencing factors to deeply explore and determine the main influencing factors of various energy consumption and the specific impact extent of these factors on various energy consumption. This study adopts the stepwise strategy, which is a combination of the forward and backward strategies. It makes each independent variable enter the model and then re-evaluates whether there are independent variables that can be eliminated. Therefore, the stepwise screening strategy provides an opportunity to eliminate insignificant independent variables at each stage of introducing independent variables.

After estimating the parameters in the model and obtaining a definite regression equation, it is necessary to test whether the regression equation truly reflects the statistical relationship between variables in general and whether the regression equation can be used for prediction, which mainly includes the goodness-of-fit test of the regression equation, the significance test of the regression equation and the significance test of the regression coefficient. The goodness-of-fit test of the regression equation is used to test how densely the sample data points are clustered around the regression line, thus evaluating how well the regression equation represents the sample data. The significance test of the regression equation is used to test whether the linear relationship between the dependent variables and all independent variables is significant. The significance test of regression coefficients is used to investigate whether there is a significant linear relationship between each independent variable and the dependent variable, that is, to investigate whether each independent variable can effectively explain the linear variation of the dependent variable. The following are the results of the regression analysis using electricity consumption, natural gas consumption, and GHG emissions as the dependent variables and combined with the final determined independent variables. In addition, Table 4 shows the results of the significance tests for the regression coefficients of the three models.

**Table 4:** The significance test results of the regression coefficients of the three models.

	Electricity Consumption Model				Natural Gas Consumption Model				GHG Emission Model			
	Non-normalized Coefficient	Normalized Coefficient	t	P-Value	Non-normalized Coefficient	Normalized Coefficient	t	P-Value	Non-normalized Coefficient	Normalized Coefficient	t	P-Value
	B	Beta			B	Beta			B	Beta		
(Constant)	236531.665		17.494	0.000*	39431.766		19.221	0.000*	98737.927		27.002	0.000*
Total Indoor Space	6.556	0.869	94.830	0.000*	1.169	0.828	76.321	0.000*	2.406	0.859	88.133	0.000*
Weekly Average Hours	−1177.093	−0.052	−6.997	0.000*	–	–	–	–	–	–	–	–
Enrollment	1426.894	1.146	27.198	0.000*	213.813	0.917	17.928	0.000*	543.445	1.176	25.564	0.000*
Number of Students Whose First Language Is Not English	−156.634	−0.070	−6.215	0.000*	−17.582	−0.042	−3.061	0.002	−51.536	−0.062	−5.034	0.000*
Number of Students Receiving Special Education Services	−3145.023	−0.521	−24.853	0.000*	−484.971	−0.429	−16.861	0.000*	−1206.279	−0.538	−23.529	0.000*
Number of School-Aged Children Who Live in Low-Income Households	−434.112	−0.079	−7.771	0.000*	−68.027	−0.066	−5.345	0.000*	−207.874	−0.102	−9.163	0.000*
Student Learning Ability	−2699.546	−0.749	−28.637	0.000*	−475.106	−0.704	−22.194	0.000*	−1164.805	−0.871	−30.527	0.000*

- the independent variable is excluded from the multiple regression model, *P-value < 0.001.

The regression equations for electricity consumption, natural gas consumption, and GHG emissions are shown in Equations (8), (9), (10), respectively. The adjusted R^2^ values for each of the three regression equations are 0.784, 0.680, and 0.741, respectively, and the observed values of the F-statistics for the significance test of the regression equations are 2287.577, 1560.257, and 2104.551, respectively, with the corresponding probability P values all less than 0.001. This indicates that all three models fit well and that the null hypothesis (i.e., there is no significant linear relationship between all independent variables and dependent variables) of the significance test of the regression equation should be rejected. It can be considered that there is a significant linear relationship between all the independent variables and the three dependent variables (electricity consumption, natural gas consumption, and GHG emissions). In addition, the regression equations show that the total indoor space and enrollment are positively related to electricity consumption, natural gas consumption, and GHG emissions. This is mainly because larger indoor spaces have more energy-consuming devices, and larger indoor spaces often have a single energy-consuming device using more power to meet the needs of users and the indoor environment, such as high-power lighting and cooling and heating equipment to meet light and indoor thermal comfort needs, resulting in an increase in energy consumption and GHG emissions. More enrollment means more students, which will lead to more energy-consuming behavior and an increase in the use frequency of high-energy-consuming equipment and greater energy consumption and GHG emissions.

The regression equation also shows that the number of students whose first language is not English, the number of students receiving special education services, the number of school-aged children who live in low-income households, and the student learning ability are inversely related to electricity consumption, natural gas consumption, and GHG emissions. This indicates that students from non-English speaking countries, students receiving special education and students from low-income households will consume less energy in their daily educational activities. In addition, the weekly average number of hours is inversely related to electricity consumption. This may be because the weekly average hours variable in the original data only reflects school hours, while during non-school hours, the energy-consuming equipment within the school tends to run continuously, thus creating the illusion that the longer the weekly average hours are, the lower the electricity consumption.

According to the normalized coefficients in the multiple regression model in Table 4, the importance of different independent variables on electricity consumption, natural gas consumption, and GHG emissions can be compared. The results show that enrollment has the largest positive impact on electricity consumption, natural gas consumption, and GHG emissions, followed by total indoor space. This indicates that compared with the increase in the total indoor space, the increase in the number of students will lead to more energy consumption and GHG emissions. This is mainly because the increase in the number of students will lead to an increase in various energy-use behaviors and an increase in the use frequency of energy-consuming equipment, which in turn leads to an increase in various types of energy consumption and GHG emissions. At the same time, this further shows that, rather than the building itself, more attention should be given to the occupants of the building to explore the energy-saving potential of the building as a result of their energy use behavior and energy use frequency. Student learning ability has the largest negative impact on electricity consumption, natural gas consumption, and GHG emissions, followed by the number of students receiving special education services. This shows that the improvement of students' learning ability is more likely to lead to the reduction of various energy consumption and GHG emissions. This is mainly because students with strong learning abilities are often more accepting of new ideas, so they will have a stronger awareness of energy conservation and environmental protection, thereby reducing their high-energy-consuming behavior. This can also further reflect the energy-saving awareness of building occupants, as well as the huge impact of energy-use behavior determined by the awareness of the building’s energy-saving potential.

(8) Y_1_ = 6.556X_1_ − 1177.093X_2_ + 1426.894X_3_ − 156.634X_4_ − 3145.023X_5_ − 434.112X_6_ − 2699.546X_7_ + 236531.665 (R^2^ = 0.784, F = 2287.577, P = 0.000)

(9) Y_2_ = 1.169X_1_ + 213.813X_3_ − 17.582X_4_ − 484.971X_5_ − 68.027X_6_ − 475.106X_7_ + 39431.766 (R^2^ = 0.680, F = 1560.257, P = 0.000)

(10) Y_3_ = 2.406X_1_ + 543.445X_3_ − 51.536X_4_ − 1206.279X_5_ − 207.874X_6_ − 1164.805X_7_ + 98737.927 (R^2^ = 0.741, F = 2104.551, P = 0.000)

### 3.2 Energy consumption differences in different types and levels of school systems

To further compare and explore the differences in different types of energy consumption and GHG emissions among different types and levels of school systems, this study used the Mann‒Whitney *U* test for corresponding analysis. The results are shown in Table 5, where the mean difference in energy consumption for different types of school systems represents the difference in various types of energy consumption and GHG emissions of Catholic school systems minus those of public school systems, and the mean difference in energy consumption for different levels of school systems represents the difference in various types of energy consumption and GHG emissions of elementary school systems minus those of secondary school systems.

**Table 5:** Mann-Whitney *U* test results of school energy consumption and GHG emissions for different types and levels of school system.

		Case number	Mean rank	Sum of ranks	Mean difference in energy consumption	Z	P-Value
School Type	Electricity Consumption (kWh)	2471	3648.15	9,014,584	−846.99	−0.701	0.483
		4873	3684.85	17,956,257			
	Natural Gas Consumption (m^3^)	2471	3422.72	8,457,542	−11787.21	−7.190	0.000∗
		4873	3799.16	18,513,299			
	GHG Emissions (kg)	2471	3414.49	8,437,201	−22852.70	−7.426	0.000∗
		4873	3803.33	18,533,640			
School Level	Electricity Consumption (kWh)	6213	3406.16	21,162,448	−365538.67	−25.232	0.000∗
		1131	5135.63	5,808,392			
	Natural Gas Consumption (m3)	6213	3421.70	21,259,008	−61507.33	−23.762	0.000∗
		1131	5050.25	5,711,832			
	GHG Emissions (kg)	6213	3407.58	21,171,298	−128387.01	−25.097	0.000∗
		1131	5127.80	5,799,542			

∗ P-value<0.001.

As seen from the table, there is no significant difference in electricity consumption among different types of school systems, but there are significant differences in natural gas consumption and GHG emissions. Meanwhile, the mean differences in natural gas consumption and GHG emissions of Catholic schools and public schools are −11,787.21 m^3^ and −22,852.70 kg, respectively, indicating that the natural gas consumption and GHG emissions of Catholic schools are lower than those of public schools. This is mainly because Catholic schools tend to admit only children of Catholic families, so their average number of students (423) is smaller than that of public schools (466), and they have a smaller average indoor space than public schools (Catholic schools and public schools are 58,989.99 square feet and 65,429.08 square feet, respectively). This further verifies the conclusion that total indoor space and enrollment are proportional to natural gas consumption and GHG emissions in the multiple regression analysis above.

In addition, there are significant differences in electricity consumption, natural gas consumption, and GHG emissions among different levels of school systems. The mean differences in electricity consumption, natural gas consumption, and GHG emissions of elementary school and secondary school are −365,538.67 kWh, −61,507.33 m^3^, and −128,387.01 kg, respectively, which shows that the electricity consumption, natural gas consumption, and GHG emissions of elementary school are lower than those of secondary school. This is due to the fact that the elementary school’s average number of students (379) and indoor space (54,521.57 square feet) are lower than the secondary school’s average number of students (863) and indoor space (111,280.02 square feet).

### 3.3 The influence of enrollment in different grades on energy consumption in different types and levels of school systems

According to the results of the multiple regression analysis above, enrollment has the greatest positive impact on electricity consumption, natural gas consumption, and GHG emissions. Therefore, under the premise of controlling the linear effects of other variables on various types of energy consumption and GHG emissions, this part uses partial correlation analysis to further explore which grade of students has a greater impact on these variables in different types and levels of school systems. The results are shown in Table 6. It can be seen from the table that there are statistically significant partial correlations between the students' enrollment in each grade and electricity consumption, natural gas consumption, and GHG emissions, both in the Catholic school system and the public school system. Among them, the electricity consumption of Catholic schools and public schools has the highest partial correlation with grade 10 (0.388) and grade 11 (0.380) respectively. The natural gas consumption of Catholic school and public school both has the highest partial correlation with grade 12, 0.191 and 0.270 respectively. And the GHG emissions of Catholic schools and public schools also both have the highest partial correlation with grade 12, 0.230 and 0.273 respectively.

**Table 6:** Partial correlation results between the students' enrollment in different grades and various energy consumption and GHG emissions.

			Electricity Consumption (kWh)		Natural Gas Consumption (m^3^)		GHG Emissions (kg)	
			Catholic School	Public School	Catholic School	Public School	Catholic School	Public School
Elementary School	Grade 1 Enrollment	Pearson correlation	0.217∗	0.282∗	0.149∗	0.225∗	0.147∗	0.226∗
		Sig.	0.000	0.000	0.000	0.000	0.000	0.000
	Grade 2 Enrollment	Pearson correlation	0.229∗	0.281∗	0.151∗	0.214∗	0.149∗	0.214∗
		Sig.	0.000	0.000	0.000	0.000	0.000	0.000
	Grade 3 Enrollment	Pearson correlation	0.245∗	0.293∗	0.165∗	0.217∗	0.165∗	0.219∗
		Sig.	0.000	0.000	0.000	0.000	0.000	0.000
	Grade 4 Enrollment	Pearson correlation	0.255∗	0.295∗	0.164∗	0.217∗	0.166∗	0.220∗
		Sig.	0.000	0.000	0.000	0.000	0.000	0.000
	Grade 5 Enrollment	Pearson correlation	0.241∗	0.294∗	0.163∗	0.211∗	0.163∗	0.214∗
		Sig.	0.000	0.000	0.000	0.000	0.000	0.000
	Grade 6 Enrollment	Pearson correlation	0.245∗	0.234∗	0.159∗	0.198∗	0.163∗	0.198∗
		Sig.	0.000	0.000	0.000	0.000	0.000	0.000
	Grade 7 Enrollment	Pearson correlation	0.296∗	0.217∗	0.175∗	0.203∗	0.190∗	0.199∗
		Sig.	0.000	0.000	0.000	0.000	0.000	0.000
	Grade 8 Enrollment	Pearson correlation	0.291∗	0.214∗	0.173∗	0.201∗	0.188∗	0.196∗
		Sig.	0.000	0.000	0.000	0.000	0.000	0.000
Secondary School	Grade 9 Enrollment	Pearson correlation	0.352∗	0.325∗	0.145∗	0.202∗	0.185∗	0.204∗
		Sig.	0.000	0.000	0.000	0.000	0.000	0.000
	Grade 10 Enrollment	Pearson correlation	0.388∗	0.362∗	0.181∗	0.237∗	0.222∗	0.241∗
		Sig.	0.000	0.000	0.000	0.000	0.000	0.000
	Grade 11 Enrollment	Pearson correlation	0.384∗	0.380∗	0.180∗	0.251∗	0.222∗	0.258∗
		Sig.	0.000	0.000	0.000	0.000	0.000	0.000
	Grade 12 Enrollment	Pearson correlation	0.382∗	0.357∗	0.191∗	0.270∗	0.230∗	0.273∗
		Sig.	0.000	0.000	0.000	0.000	0.000	0.000

∗ P-value<0.001.

**Fig. 1:**
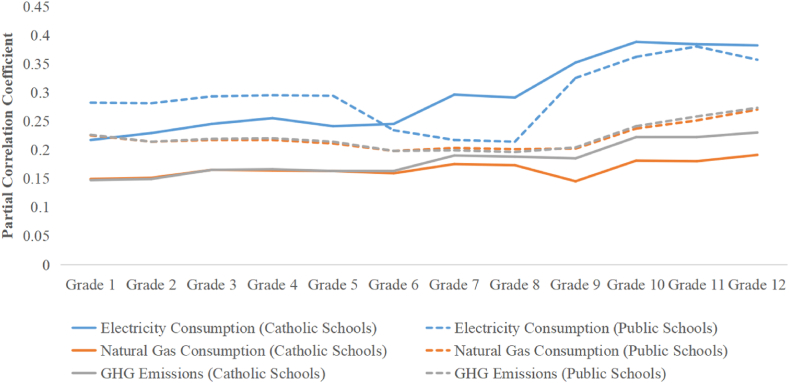
The variation trend of the partial correlation coefficient with grade.

Meanwhile, the partial correlation between student enrollment in each grade and various energy consumption and GHG emissions has a trend of gradually increasing as the grade level increases in Catholic elementary schools, while in public elementary schools, there is a trend of gradually decreasing with the increase in grade level (see Fig. 1). This shows that in public elementary schools, the partial correlation between student enrollment in lower grades (grades 1–5) and various energy consumption and GHG emissions is slightly higher than that in upper grades (grades 6–8), mainly because in public elementary school, lower grade students require more care, thus increasing their unit energy consumption. While in Catholic elementary school, this phenomenon does not appear, mainly because Catholic elementary school generally only enrolls Catholic students, thus Catholic elementary school has some extra religious courses besides regular education courses, such as religious prayer (especially in upper grades), which gives students more opportunities to stay indoors and generate more energy and offsets the reduced energy consumption of the upper-grade students because they do not need extra care.

In addition, the partial correlation between student enrollment in each grade and various energy consumption and GHG emissions has a trend of gradually increasing as the grade level increases in both Catholic secondary school and public secondary school (see Fig. 1). This is mainly because with the increase of grade, the number of courses increases, which increases the learning of high-energy-consuming courses such as computer studies, and thus lead to an increase in the energy consumption of a single student.

## 4 Discussion

Obviously, the number of student enrollments has the greatest positive impact on electricity consumption, natural gas consumption, and GHG emissions. While this study is more focused on the student backgrounds which may disclose more social factors that need to be considered in energy policy. This study found that student learning ability has the greatest negative impact on energy consumption in elementary and secondary schools, followed by the number of students receiving special education services. This means that the difference in students' education level and learning ability will have an impact on energy consumption, which is mainly because students with strong learning abilities can receive a better education. Therefore, energy conservation and environmental protection awareness will be stronger and will consciously reduce their high energy-use behaviors and energy-use frequencies. This finding has also been corroborated by other studies; for example, Pisello et al. [43] argue that educational background promotes environmental protection awareness and that higher levels of environmental awareness may lead people to adopt resilient energy-saving behavior. It is important to develop measures to enhance energy conservation education so that more people are informed and aware of energy conservation and environmental protection [44], which will promote an increase in energy-conservation behaviors.

In Canadian educational system, gifted program students are often required to do many reading and extracurricular activities to develop their multiple skills, and these classes and outdoor activities may not be energy intensive. This finding has also been confirmed by studies such as Buldur et al. (2020) who used a mixed qualitative and quantitative approach to investigate the impact of nature education programs on secondary school students' perceptions of renewable energy and showed that such programs made a significant contribution to increasing students' perceptions of renewable energy. Arts program students often focus more on their artistic skills and use less high-energy-consuming equipment such as computers, also resulting in lower energy consumption. In addition, these students are likely to have a better understanding of new ideas, concepts, and trends and are therefore more likely to be more environmentally conscious, resulting in less high-energy-consuming behavior in their daily school life.

The number of students whose first language is not English, the number of students who receive special education services, the number of school-aged children who live in low-income households, have a negative impact on energy consumption. In other words, school with more students from the disadvantaged groups consume less energy, which arouses attention to their school activities. On one hand, students from those groups have lower energy consumption because they are more likely to adopt energy-saving behaviors due to their family’s financial situation or frugal lifestyles, and these behaviors are often retained during school life, resulting in lower levels of energy consumption. On the other hand, it indicates that these schools may have less intensive learning activities compared to other schools. Energy consumption is a reflection of education activities and learning duration, and the energy saving should not compromise a fair and just education system for the disadvantaged groups.

The development of energy-saving policies and programs to improve energy efficiency is an important approach to managing energy consumption in schools [22], and the views of policy makers are critical to the effective implementation of policies and programs [45]. Together, the results of these studies show that student background characteristics and learning activities have a significant impact on school energy consumption as important factors that are not reflected in many existing policies and programs. As a typical public product, schools are the second largest sector of public infrastructure spending after highways [46], requiring significant financial and resource support from the government, and the high cost of energy consumption accounts for a significant portion of the investment. Against this backdrop, it is more important that we take a holistic and cautious attitude when formulating policies related to energy consumption in schools. The policy makers should break the shackles of the focus on the characteristics of the school facilities and set the content and ultimate purpose in a way that is appropriate to the characteristics of the students (including their family background, their learning ability, etc.) of schools in different regions and at different levels. Reducing costs by constantly optimizing energy-saving policies and programs will lead to a greater allocation of investment in schools to education itself in order to adequately meet the development of students.

## 5 Conclusion

This study takes Ontario, Canada, as the study area and investigates the school energy consumption data, school characteristic data, and student information data of 3672 elementary and secondary schools in the region in 2018 and 2019. A series of statistical methods are used to analyze the influencing factors (including students' background, learning ability, enrollment, and total indoor space) on energy consumption and their importance on energy consumption. At the same time, the differences in energy consumption in different types and levels of school systems are also analyzed, and further explore which grade of students has a greater impact on various types of energy consumption and GHG emissions in different types and levels of school systems.

The number of students whose first language is not English, the number of students receiving special education services, the number of school-aged children who live in low-income households, and student learning ability are all inversely proportional to electricity consumption, natural gas consumption, and GHG emissions, of which student learning ability has the largest negative impact. The findings imply for developing effective programs for encouraging environmental-friendly learning activities while special attention paid to the disadvantaged groups who should receive fair education under any energy saving policy.

In addition, there are significant differences in electricity consumption, natural gas consumption, and GHG emissions among different levels of school systems. The partial correlations between student enrollment in each grade and electricity consumption, natural gas consumption, and GHG emissions are statistically significant in different school systems. Among them, the partial correlation between student enrollment in each grade and energy consumption and GHG emissions has a trend of gradually increasing as the grade levels increase in Catholic elementary schools, Catholic secondary schools, and public secondary schools, while it has a trend of gradually decreasing with the increase in grade levels in public elementary school. These findings help policy-makers find more specific energy-saving potential in the school systems considering the differences existing among different grade levels.

This study contributes to the research of energy consumption in the education sector where universities have been the main research subject, while elementary and secondary schools are missing. Meanwhile, it also introduces the demographic attributes into energy consumption analysis for the first time, and takes into account the impact of students' backgrounds on energy consumption in elementary and secondary schools aiming to identify the energy saving potential of students with different backgrounds. Relevant conclusions are conducive to formulating and carrying out targeted energy saving education for different students. In addition, this study highlights that energy is a natural resource and the consumption of energy reflects the education resource distribution. This paper explores the energy consumption of different types and levels of school systems, as well as the differences among some disadvantaged groups, such as students from low-income families, and finds that their energy distribution is uneven. This suggests that policy makers should appropriately consider disadvantaged groups when making energy policies or electricity pricing policies and that energy saving shall not compromise their needs of learning activities.

This study also has some limitations. This study only investigated several key factors to look at student background conditions while missing out school curriculum conditions due to data availability; schools with similar student characteristics may have different educational activities and consequently exhibit different energy consumption levels. In addition, this paper also does not consider the impact of students' personality characteristics on school energy consumption due to the difficulty of measurement and quantification. Another limitation is about the time period which mainly covers 2017.9–2020.6. The energy consumption and demographic data after that are not covered; this is not just because of data unavailability but also due to the impact of the pandemic which may bias the study. Therefore, more factors should be investigated in the future to further refine the study. At the same time, the differences in the specific energy-use behavior of students with different characteristics can be investigated to explain in more details about the differences found between high-income and low-income students, students with higher and lower learning abilities, and students receiving special and normal educations.

## Author contribution statement

Zefeng Huang: Conceived and designed the experiments; Performed the experiments; Analyzed and interpreted the data; Contributed reagents, materials, analysis tools or data; Wrote the paper.

Zhonghua Gou: Conceived and designed the experiments; Contributed reagents, materials, analysis tools or data; Wrote the paper.

Senhong Cai: Contributed reagents, materials, analysis tools or data; Wrote the paper.

## Data availability statement

Data will be made available on request.

## Declaration of competing interest

The authors declare that they have no known competing financial interests or personal relationships that could have appeared to influence the work reported in this paper
